# Classical scrapie prions are associated with peripheral blood monocytes and T-lymphocytes from naturally infected sheep

**DOI:** 10.1186/s12917-016-0651-6

**Published:** 2016-02-04

**Authors:** Rohana P. Dassanayake, Sally A. Madsen-Bouterse, Thomas C. Truscott, Dongyue Zhuang, Michelle R. Mousel, William C. Davis, David A. Schneider

**Affiliations:** Department of Veterinary Microbiology and Pathology, College of Veterinary Medicine, Washington State University, Pullman, WA 99164-6630 USA; Animal Disease Research Unit, Agricultural Research Service, U.S. Department of Agriculture, Pullman, WA 99164-6630 USA

**Keywords:** Bioassay, Classical scrapie, Lambs, Monocytes, T lymphocytes

## Abstract

**Background:**

Classical scrapie is a transmissible spongiform encephalopathy (TSE) that affects sheep and goats. Our previous bioassay studies in lambs revealed that scrapie prions could be detected in association with peripheral blood monocular cells (PBMC), B lymphocytes and platelet-rich plasma fractions. In the present study, bioassay in lambs was again used to determine if scrapie prions are associated with the other two subsets of PBMC, monocytes and T lymphocytes.

**Results:**

PBMC, monocytes and T lymphocytes were isolated from two preclinically affected VRQ/VRQ sheep naturally infected with classical ovine scrapie and intravenously transfused into VRQ/VRQ lambs post-weaning. As determined using standard immunohistochemistry for scrapie, abnormal isoforms of prion protein were detected in lymphoid tissues of lambs inoculated with PBMC (4/4 recipient lambs), monocytes (2/5) and T lymphocytes (1/4). Prion protein misfolding activity was detected by serial protein misfolding cyclic amplification (sPMCA) in PBMC from monocyte and T lymphocyte recipient sheep in agreement with antemortem rectal biopsy results, but such prion protein misfolding activity was not detected from other recipients.

**Conclusions:**

These findings show that scrapie prions are associated with monocytes and T lymphocytes circulating in the peripheral blood of sheep naturally infected with classical scrapie. Combined with our previous findings, we can now conclude that all three major subsets of PBMC can harbor prions during preclinical disease and thus, present logical targets for development of a sensitive assay to detect scrapie prions. In this regard, we have also demonstrated that sPMCA can be used to detect scrapie prions associated with PBMC.

## Background

Prion diseases or transmissible spongiform encephalopathies (TSEs) are fatal and chronic neurodegenerative disorders that affect a variety of species. Scrapie is the naturally occurring form that occurs in domestic sheep and goats. The infectious agent, a prion, consists primarily of an abnormal conformational isoform (PrP^Sc^) of normal cellular prion protein (PrP^C^) [1, 2]. Classical scrapie is characterized by the accumulation of PrP^Sc^ in the central nervous system and, in most cases, lymphoid tissues [3, 4]. Antemortem diagnosis of scrapie infections in sheep can be performed by immunohistochemical analysis of rectal tissues [5, 6] and nictitating membranes [7] where infected animals show PrP^Sc^ accumulation in the lymphoid follicles.

Our previous bioassay studies in lambs and a transgenic mouse line (Tg338) expressing the ovine VRQ *PRNP* allele revealed that scrapie prions were associated with different peripheral blood components such as buffy coat, peripheral blood mononuclear cells (PBMC), B lymphocytes and platelet-rich plasma from preclinically and clinically affected sheep naturally infected with classical scrapie [8, 9]. The association of prion infectivity with all the PBMC subsets and platelet-rich plasma from sheep experimentally inoculated with the PG127 classical scrapie isolate has also been shown using Tg338 mice [10]. However, a TSE- ELISA based study concluded that PrP^Sc^ in scrapie affected sheep blood (non-PG127 scrapie isolates) was principally associated with a subpopulation of B lymphocytes but not with monocytes or T lymphocytes [11].

Animal bioassay is one of the most sensitive systems for detection of prions as was evident from our previous work with the lamb bioassay model [8, 9] where classical scrapie prions were detected in blood from both preclinically and clinically affected sheep naturally infected with classical ovine scrapie isolates despite a lack of detection by the TSE-ELISA. Therefore, to determine whether monocytes and T lymphocytes prepared from sheep naturally infected with classical ovine scrapie harbor scrapie prions, we again used this sensitive VRQ/VRQ lamb bioassay model.

## Results

### Lambs transfused with PBMC, monocytes or T lymphocytes developed scrapie

**Fig. 1:**
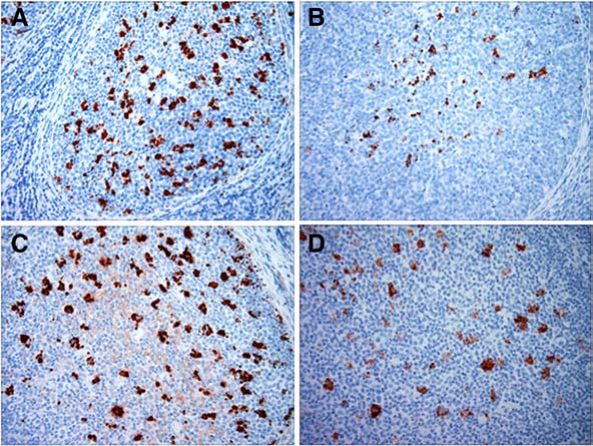
Detection of PrP^Sc^ immunolabeling in the follicles of rectoanal mucosa-associated lymphoid tissues of donor and recipient sheep. Note the PrP^Sc^ immunolabeling (dark red) was visible in the RAMALT follicles of donor sheep (**a**, animal ID: 4454) and recipient sheep transfused with PBMC (**b**, animal ID: 4601), monocytes (**c**, animal ID: 4595) or pan T lymphocytes (**d**, animal ID: 4590). IHC was performed using a mixture of prion mAbs F99/97.6.1 and F89/160.1.5. (2.5 μg/mL each) and AEC chromogen

Preclinical stages of scrapie infection in two VRQ/VRQ donor sheep naturally exposed to classical ovine scrapie were determined before blood sample collection. Strong PrP^Sc^ immunolabeling in the rectoanal mucosa-associated lymphoid tissue (RAMALT) follicles from both donor sheep confirmed preclinical scrapie infection (Fig. 1a, Table 1). As a positive control, VRQ/VRQ recipient lambs in treatment group 1 were transfused with PBMC prepared from the donor sheep. Estimates of the total number of PBMC transfused into each recipient lamb are listed in Table 2. Transmission of scrapie infection was evident as PrP^Sc^ immunolabeling was detected in RAMALT follicles from one of the four recipient lambs when biopsied at 6 months post inoculation (mpi) (Fig. 1b, Table 2), thus confirming presence of scrapie prions in donor sheep blood at the time of blood collection for the inoculation. Recipients and uninoculated control animals were humanely euthanized at 10 mpi and tissues were collected at necropsy for immunohistochemistry (IHC). PrP^Sc^ immunolabeling was visible in RAMALT follicles, alimentary tract-associated and peripheral lymphoid tissues of all four recipient sheep but PrP^Sc^ immunolabeling was not detected in the brains of any recipients (Table 2). PrP^Sc^ immunolabeling was not detected in any of the tissues examined from two uninoculated control sheep co-housed with recipient sheep (Table 2).

**Table 1:** Blood donor information

Donor ID	*PRNP*	Scrapie status^a^	Age blood collected (in months)	Age first scrapie clinical signs (in months)	Age at necropsy (in months)	Postmortem PrP^Sc^ detection by IHC	
						Lymphoid tissues^b^	Brains
4438	VRQ/VRQ	Preclinical	18	24	25	(+)	(+)
4454	VRQ/VRQ	Preclinical	18	25	25	(+)	(+)

*Abbreviations*: *PRNP* prion genotype, *IHC* Immunohistochemistry, (+), Positive for PrP^Sc^ immunolabeling; ^a^Preclinical scrapie status of the animals were identified by the detection of PrP^Sc^ immunolabeling in the rectal tissues using a mixture of prion mAbs F99/97.6.1 and F89/160.1.5 by immunohistochemistry; ^b^Lymphoid tissues included alimentary tract-associated lymphoid tissues (tonsils, retropharyngeal, mesenteric such as ruminal, abomasal, duodenal, jejunal, ileocecal, distal ileum, ileocecal junction lymph nodes, ileum, and spleens) and peripheral lymph nodes (prescapular, prefemoral, and popliteal)

**Table 2:** Summary of the scrapie transmission results in sheep transfused with PBMC, monocytes or T lymphocytes from preclinical sheep with classical ovine scrapie infection

Group	Donor ID	Cell type	Cell number^a^	Lamb *PRNP*			PrP^Sc^ detection by IHC^b^											sPMCA	
							Antemortem					Postmortem						Antemortem	
				Recipients	Controls	Animal ID	Rectal tissues					Rectal tissues		Other lymphoid tissues^c^		Brains		PBMC	
							4	6	12	18	21	10	25	10	25	10	25	15	17^#^
1	4454	PBMC	1.3 × 10^8^	VRQ/VRQ		4599	(−)	(−)	NA	NA	NA	(+)	NA	(+)	NA	(−)	NA	NA	NA
				VRQ/VRQ		4601	(−)	(+)	NA	NA	NA	(+)	NA	(+)	NA	(−)	NA	NA	NA
	4438		4.5 × 10^8^	VRQ/VRQ		4598	(−)	(−)	NA	NA	NA	(−)	NA	(+)	NA	(−)	NA	NA	NA
				VRQ/VRQ		4600	(−)	(−)	NA	NA	NA	(+)	NA	(+)	NA	(−)	NA	NA	NA
			NA		VRQ/VRQ	4602	(−)	(−)	NA	NA	NA	(−)	NA	(−)	(−)	(−)	NA	NA	NA
					VRQ/VRQ	4603	(−)	(−)	NA	NA	NA	(−)	NA	(−)	(−)	(−)	NA	NA	NA
2	4454	Monocytes	2.2 × 10^6^	VRQ/VRQ		4594	(−)	ND	(−)	(−)	(−)	NA	(−)	NA	(−)	NA	(−)	(−)	ND
				VRQ/VRQ		4595	(−)	ND	(+)	(+)	(+)	NA	(+)	NA	(+)	NA	(+)	(P)	ND
	4438		4.5 × 10^6^	ARQ/VRQ		4546	(−)	ND	(−)	(−)	(−)	NA	(−)	NA	(−)	NA	(−)	(−)	ND
				VRQ/VRQ		4593	(−)	ND	(−)	(−)	(−)	NA	(−)	NA	(−)	NA	(−)	(−)	ND
				VRQ/VRQ		4597	(−)	ND	(+)	(+)	(+)	NA	(+)	NA	(+)	NA	(+)	(P)	ND
			NA		VRQ/VRQ	4596	(−)	ND	(−)	(−)	ND	NA	(−)	NA	(−)	NA	(−)	(−)	ND
3	4454	T lymphocytes	5.9 × 10^6^	VRQ/VRQ		4539	(−)	ND	(−)	(−)	(−)	NA	(−)	NA	(−)	NA	(−)	ND	(−)
				VRQ/VRQ		4590	(−)	ND	(−)	(+)	(+)	NA	(+)	NA	(+)	NA	(+)	ND	(P)
	4438		9.9 × 10^6^	VRQ/VRQ		4588	(−)	ND	(−)	(−)	(−)	NA	(−)	NA	(−)	NA	(−)	ND	(−)
				VRQ/VRQ		4589	(−)	ND	(−)	(−)	(−)	NA	(−)	NA	(−)	NA	(−)	ND	(−)
			NA		VRQ/VRQ	4591	(−)	ND	(−)	(−)	ND	NA	(−)	NA	(−)	NA	(−)	ND	(−)
					VRQ/VRQ	4592	(−)	ND	(−)	(−)	ND	NA	(−)	NA	(−)	NA	(−)	ND	(−)

*Abbreviations*: *PRNP* prion genotype, *PBMC* peripheral blood mononuclear cells, *NA* not applicable, *ND* Not done, *sPMCA* Serial protein misfolding cyclic amplification, *P* positive for prion protein misfolding activity; ^#^months post-inoculation; (+) = PrP^Sc^ immunolabeling was detected; (−) = PrP^Sc^ immunolabeling was not detected; ^a^indicate the total number of PBMC, monocytes or T lymphocytes isolated from a 50 mL whole blood sample volume; ^b^PrP^Sc^ immunolabeling in the tissues were detected using a mixture of prion mAbs F99/97.6.1 and F89/160.1.5 by immunohistochemistry; ^c^Other lymphoid tissues include alimentary tract-associated lymphoid tissues (tonsils, retropharyngeal, mesenteric such as ruminal, abomasal, duodenal, jejunal, ileocecal, distal ileum, ileocecal junction lymph nodes, ileum, and spleens) and peripheral lymph nodes (prescapular, prefemoral, and popliteal)

Four VRQ/VRQ lambs and one ARQ/VRQ lamb in group 2 were intravenously transfused with the monocytes prepared from the donor sheep (Table 2). PrP^Sc^ accumulation in RAMALT follicles was not detected in any of the recipient lambs at 4 mpi. PrP^Sc^ accumulation was detected in RAMALT follicles from two of the five recipient animals (4595 and 4597) at 12 mpi (Fig. 1c, Table 2), but was not detected from the other three recipients at 15, 18 or 21 mpi. All six animals were humanely euthanized at 25 mpi. PrP^Sc^ immunolabeling was visible in RAMALT follicles, alimentary tract-associated and peripheral lymphoid tissues as well as brains (at the levels of obex) from the same previously rectal biopsy positive two animals (Table 2). PrP^Sc^ immunolabeling was not detected in any of the tissues examined from the remaining three recipient sheep and uninoculated control sheep (Table 2).

Four VRQ/VRQ lambs in group 3 were intravenously transfused with T lymphocytes prepared from the same donor sheep. PrP^Sc^ immunolabeling was not detected in RAMALT follicles of any of the recipient lambs when biopsied at 4, 12, and 15 mpi. PrP^Sc^ immunolabeling was detected in RAMALT follicles from one of the four recipients (4590) when biopsied at 18 mpi (Fig. 1d, Table 2), but was not detected from the other three recipients at 21 mpi. All six animals in group 3 were humanely euthanized at 25 mpi. PrP^Sc^ immunolabeling was detected in all the tissues including the brain (at the levels of obex) from the previously rectal biopsy positive animal, whereas PrP^Sc^ immunolabeling was not detected in any of the tissues examined from the remaining three recipient sheep as well as two uninoculated control sheep (Table 2).

### Prion protein misfolding activity was detected in PBMC of monocyte and T lymphocyte recipient sheep

**Fig. 2:**
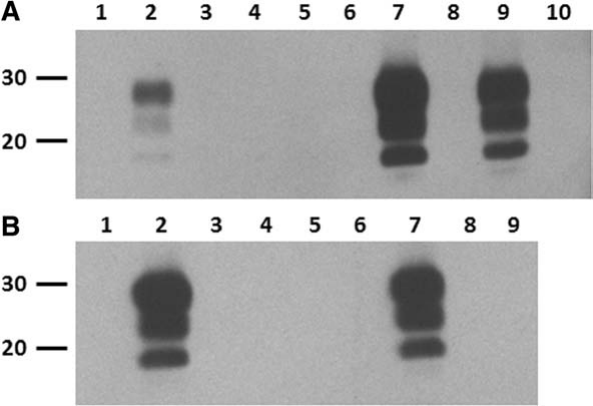
Prion protein misfolding activity detected by sPMCA in PBMC of recipient sheep transfused with monocytes or T lymphocytes from scrapie-affected sheep. PBMC (1x10^7^ cells) were added to mNBH and subjected to six PMCA rounds of 48 cycles of sonication and incubation. Samples were diluted 1:3 into fresh mNBH between rounds. Pre- and post-PMCA samples underwent proteinase K digestion (200 μg/ml for 90 min at 37 °C) prior to western blot analysis with prion mAb P4. Representative western blots are shown. **a** PBMC prepared from positive control scrapie sheep (4124) pre- and post-sPMCA (lanes 1 and 2, respectively). PBMC prepared from monocyte recipient sheep (lane 4 = 4546, lane 5 = 4593, lane 6 = 4594, lane 7 = 4595, lane 9 = 4597) and an uninoculated control sheep (lane 8 = 4596). A scrapie naïve sheep (lane 3 = 4545) and mNBH (lane = 10) served as negative controls for sPMCA. **b** PBMC from positive control scrapie sheep (4124) is shown pre- and post-sPMCA (lanes 1 and 2, respectively). PBMC prepared from T lymphocyte recipient sheep (lane 4 = 4539, lane 5 = 4588, lane 6 = 4589, lane 7 = 4590) and uninoculated control sheep (lane 8 = 4591, lane 9 = 4597); and mNBH (negative sPMCA control, lane = 3). Molecular mass markers (in kDa) are indicated on the left of the blots

Serial protein misfolding cyclic amplification (sPMCA) was performed to assess preclinical scrapie infection in recipient sheep. PBMC isolated from blood samples of monocyte (15 mpi) and T lymphocyte recipients (17 mpi) were subjected to six rounds of sPMCA as previously described [12, 13]. Consistent with the rectal biopsy results, prion protein misfolding activity (PrP^Sc^) was detected in two recipients in the monocyte group (Fig. 2a; lanes 7 = 4595 and lane 9 = 4597) and one in the T lymphocyte group (Fig. 2b; lane 7 = 4590) following sPMCA. Such prion protein misfolding activity was not observed with the remaining monocyte and T lymphocyte recipients as well as the uninoculated control animals in both groups (Table 2).

## Discussion

Using a lamb bioassay model, we have previously shown the transmission of scrapie following transfusion of PBMC, B lymphocytes and platelet-rich plasma prepared from sheep naturally infected with classical ovine scrapie [8, 9]. In the present study, we have further identified monocytes and T lymphocytes as peripheral blood cell types that harbor scrapie prions, thus demonstrating all three subsets of PBMC can transmit scrapie. Previous studies by us [14] and others [15] revealed that occasional PrP^Sc^ granules positive B and T lymphocytes were present in the lymphoid tissues of scrapie-infected sheep. Despite our best efforts, we could not detect PrP^Sc^ granules labeling in the circulating monocytes, B or T lymphocytes. Therefore, although our studies clearly confirmed that all three PBMC subsets harbor prion infectivity, we cannot conclude whether PrP^Sc^ and scrapie infectivity of PBMC subsets were actively generated or acquired passively from other cells or tissues.

Our previous transfusion studies in lambs revealed that PBMC or B lymphocytes isolated from 50 mL whole blood volumes from naturally infected sheep with scrapie was able to efficiently transmit the disease [8, 9]. Andreoletti et al. (2012) has reported that as little as 0.2 mL whole blood volume collected from scrapie PG127 isolate experimentally inoculated sheep was sufficient to transmit scrapie infection to recipient lambs [16]. Although our most recent lamb bioassay study revealed that intravenous transfusion of B lymphocyte prepared from 5 mL blood sample volume was sufficient to transmit scrapie from clinically affected sheep naturally infected with classical scrapie, only two of the three recipient lambs developed preclinical scrapie infection [9]. Since our donor sheep acquired scrapie by natural exposure routes as compared to experimental oral administration of scrapie brain homogenates in other studies, as a precautionary measure, we decided to isolate and then transfuse monocytes and T lymphocytes from 50 mL blood sample volume for the present study. For our previous transfusion studies in lambs, whole blood or several blood fractions from 10 different donor sheep preclinically or clinically infected with natural ovine scrapie were examined [8, 9]. To further expand the number of donors used for blood transfusion studies in lambs, blood samples were collected from preclinically affected two additional donor sheep naturally infected with classical ovine scrapie.

We and others have also shown that VRQ/VRQ recipient lambs produced early preclinical scrapie infections following transfusion of blood fractions from VRQ/VRQ scrapie donor sheep [8, 16]. Therefore, the present study also took advantage of this short incubation period in the natural host as model biased for quick detection of scrapie prions in blood fractions having relatively high prion titers. As expected, early scrapie transmission in PBMC recipients was detected in all four recipients by 10 mpi, confirming the presence of scrapie prions in PBMC in both sheep when prepared from a 50 mL blood volume. In contrast, transfusion of the monocyte or T lymphocyte blood cell fractions, each also prepared from 50 mL blood volumes, resulted in respective transmission rates of only two of the five and one of four recipients. Our previous blood transfusion study confirmed the efficient transmission of scrapie from VRQ/VRQ donors to ARQ/VRQ recipient sheep such that PrP^Sc^ accumulation can be detected in RAMALT follicles within 6–10 mpi [8]. A recent study by Gonzalez et al., (2014) revealed that although delayed scrapie progression was observed in ARQ/VRQ sheep compered to VRQ/VRQ at 85 dpi and 112 dpi, PrP^Sc^ accumulation profiles were indistinguishable between two *PRNP* genotypes at approximately 200 dpi [17]. Since our recipient sheep were euthanized at 25 mpi, the lack of preclinical scrapie in ARQ/VRQ recipient is very much unlikely due to the *PRNP* polymorphism at 136 codon.

The longer times to first detection of scrapie transmission and apparently lower transmission rates are likely due to relatively lower infectious scrapie titers in monocyte and T lymphocyte inocula as compared to PBMC inocula. In particular, the lack of scrapie infectivity in both T lymphocyte inocula prepared from donor sheep 4438 may have contained an insufficient dose of infectious prion since both monocyte and PBMC inocula prepared from the same donor was able to transmit scrapie infection to recipient lambs. It would have been informative to perform sPMCA on the donor cell fractions to determine the relative level of PrP^Sc^ associated with each blood cell type, which may have provided an insight into why some donor fractions did not transmit disease. For example, duplicate monocyte or T cell preparations (each from the same donor) did not induce pre-clinical scrapie disease in recipient sheep. However, since we did not collect additional blood samples from the donor sheep during the transfusion, or at necropsy, we were not able to perform sPMCA on the donor cell fractions. The total number of monocytes and T lymphocytes transfused into each lamb was in a range of 2.2–4.5 × 10^6^ and 5.9–9.9 × 10^6^, respectively. A previous study by Edwards et al., (2010) reported that PrP^Sc^ was not associated with CD14^+^ monocyte or CD2^+^ T lymphocyte but was associated with a subset of B lymphocytes [11]. The lack of PrP^Sc^ signal detected in the monocytes of scrapie-infected sheep by ELISA was attributed to the lack of scrapie prion or the suboptimal cell numbers used (less than 3 × 10^6^). However, positive PrP^Sc^ signal was not reported even after using the optimal number of CD2^+^ T lymphocytes (1 × 10^7^) [11]. Based on the findings of present bioassay study in lambs, we can suggest that although scrapie prion was clearly present in both cell types, scrapie titers in T lymphocytes may be lower as compared to PBMC. In our previous bioassay study in lambs, transmission of scrapie was confirmed in all three lambs receiving 5 × 10^6^ B lymphocytes and two of the three lambs receiving 2.5 × 10^6^ B lymphocytes by 10 mpi [9]. Although animal numbers used in this and previous studies [8, 9] are not sufficient for a powerful comparison, the findings in this study suggest that scrapie titers in similar numbers of monocytes and B lymphocytes may not be much different from each other.

Although PrP^Sc^ immunolabeling was detected in the brain tissues of monocytes and T lymphocytes recipients when necropsied at 25 mpi, such PrP^Sc^ immunolabeling was not detected in the brain tissues of PBMC recipients when necropsied at 10 mpi. The lack of PrP^Sc^ accumulation in brain tissues of PBMC recipient lambs is not surprising and most likely due to the early euthanasia at 10 mpi. Such observations in lambs have been previously reported by us [8, 9] and others [4, 18]. Lateral transmission of scrapie has been previously reported amongst lambs fed milk derived from scrapie infected sheep [19]. In our study, lateral transmission was not detected in any of the uninoculated control lambs co-housed with transfusion recipients. These observations clearly indicate that the recipient sheep developed preclinical infections only from the transfused PBMC, monocytes or T lymphocytes.

Several studies have suggested that peripheral blood leukocytes can be used as appropriate targets to detect preclinical vCJD infection in primates and BSE infection in sheep when coupled with PMCA [20, 21]. Serial PMCA has also been successfully applied to detection of prion protein misfolding activity associated with peripheral blood buffy coat cells or PBMC from the scrapie-affected sheep [10, 12]. Although sPMCA was not performed at regular intervals similar to rectal biopsies, the results of sPMCA performed on PBMC isolated from monocyte and T lymphocyte recipient sheep were concordant with scrapie IHC results performed on antemortem and postmortem lymphoid tissue samples. Although this study was not designed to determine the diagnostic accuracy (sensitivity/specificity) of sPMCA, these limited results support the use of PBMC as samples useful for the further development of highly sensitive antemortem diagnostic assay to determine scrapie infection in sheep.

## Conclusions

This study demonstrates that monocytes and T lymphocytes, in addition to B lymphocytes, harbor classical ovine scrapie prions in the blood of naturally scrapie-infected sheep. These findings also support the conclusion that PBMC might be a suitable blood fraction to target for the development of an in vitro blood-based diagnostic test to assess preclinical classical scrapie infection in sheep.

## Methods

### Scrapie blood donor sheep

All the animal experimental protocols used in this study were approved by the Institutional Animal Care and Use Committee (IACUC) at Washington State University. Two, approximately 18-month-old preclinically affected VRQ/VRQ Columbia sheep (4438 and 4454) naturally infected with classical scrapie (housed at the ARS research facility, Pullman, WA) were selected as blood donors for the present study. Both donors showed clinical signs of scrapie at approximately 25 months of age, therefore the animals were humanely euthanized (Table 1). The prion genotypes of donor and recipient sheep were determined by sequencing of the open reading frame of *PRNP* as described previously [22]. *PRNP* genotypes are shown by the deduced amino acid residues at codons 136, 154 and 171, respectively (Tables 1 and 2).

### Preparation of blood components for transfusion

Jugular venous blood was collected into 60 mL syringes containing acid citrate dextrose as the anticoagulant. A total of 300–350 mL whole blood was collected from each donor sheep. Whole blood was transferred into sterile 50 mL conical-bottom tubes and centrifuged at 380 x g for 30 min at room temperature. After plasma had been removed, the buffy coat was collected and resuspended in phosphate-buffered saline containing 2 mM EDTA (PBS-EDTA, pH 7.2). Peripheral blood mononuclear cells (PBMC) were isolated from the buffy coat suspensions using Accu-Paque^TM^ density gradient solution (Accurate Chemicals, Westbury, NY) as previously described [8]. Contaminated erythrocytes in PBMC were removed by a short incubation with erythrocyte lysis solution (Qiagen Inc., Valence, CA) followed by two washes in PBS-EDTA. PBMC were counted and resuspended in 10 mL normal saline (0.9 % NaCl, pH 7.0) and kept on ice until transfusion. A magnetic-activated cell sorting system (MACS; Miltenyi Biotech, Auburn, CA) was used to isolate specific PBMC subset populations as previously described [8, 9]. The mouse anti-caprine CD14 (CAM36A, IgG_1_) specific monoclonal antibody (mAb) which cross reacts with sheep CD14 was used to isolate monocytes [23]. The mouse anti-bovine CD2 (MUC2A, IgG_2a_) and anti-bovine γδ (BAQ4A, IgG_1_) specific mAbs which cross react with sheep CD2 (αβ) and γδ T lymphocytes were used in combination to isolate total (pan) T lymphocytes (αβ and γδ). After isolation, monocytes and pan T lymphocytes were counted, resuspended in 10 mL normal saline separately and kept on ice until the transfusion. The purities of each isolated subset of cells were above 95 % as assessed by flow cytometry (data not shown). The total numbers of PBMC, monocytes and pan T lymphocytes isolated and transfused from each initial 50 mL whole blood are listed in Table 2.

### Transfusion of blood components into lambs

Seventeen VRQ/VRQ and one ARQ/VRQ) mixed-breed (Columbia, Rambouillet, Suffolk or Targhee crosses) 5-month-old lambs were obtained from a scrapie negative flock and were divided into three groups with six lambs per group and housed in three separate indoor isolation rooms (Table 2). Within each isolation room, one or two lambs did not receive a transfusion, but rather served as negative controls. During the transfusion, lambs were physically restrained and blood components (Table 1) were administered through the jugular vein. Since animals were housed indoors, each animal received a subcutaneous injection of vitamins A and D (Agri Laboratories Ltd, St. Joseph, MO) at monthly intervals.

### Tissue sampling and immunohistochemistry

Transmission of scrapie infection to recipient lambs was confirmed by biopsy of the rectal mucosa and detection of PrP^Sc^ in rectoanal mucosa-associated lymphoid tissue (RAMALT) follicles by immunohistochemistry (IHC). Samples were collected from lambs in each group as follows: Group 1: rectal biopsy at 2, 4, and 6 months post inoculation (mpi); Groups 2 and 3: rectal biopsy at 4, 12, 15, 18 and 21 mpi. All the animals were humanely euthanized by intravenous administration of a pentobarbital-based euthanasia solution (Vortech, Dearborn, MI): group 1 at 10 mpi and groups 2 and 3 at 25 mpi. The brains, alimentary tract-associated lymphoid tissues (tonsil, retropharyngeal, ruminal, abomasal, duodenal, jejunal, ileal, ileocecal junction, and distal colon lymph nodes, ileum, rectal tissues and spleen), and peripheral lymph nodes (prescapular, prefemoral, popliteal lymph nodes) were collected at necropsy. Standard IHC technique for PrP^Sc^ in formalin-fixed paraffin embedded tissues and automated immunolabeler (Benchmark, Ventana Medical Systems, Tuscon, AZ) was used to detect scrapie transmission to recipient lambs as previously described [8, 14]. PrP^Sc^ in the tissues was identified using a mixture of prion mAbs F99/97.6.1 [24] and F89/160.1.5 [25] (2.5 μg/mL each) with basic AEC detection kits (Ventana). Samples were considered positive for PrP^Sc^ if dark red precipitates were detected in the lymphoid follicles or dorsal motor nucleus of the vagus nerve at the level of obex under bright-field microscopy.

### Serial protein misfolding cyclic amplification (sPMCA)

Prion protein misfolding activity in PBMC isolated from recipient sheep was assessed by sPMCA as described previously but with some modifications [12, 13, 26]. PBMC isolated from inoculated and uninoculated sheep in group 2 at 15 mpi and group 3 at 17 mpi were used for analysis. PBMC prepared from preclinically or clinically affected VRQ/VRQ sheep (4124 and 4125) with naturally acquired classical scrapie and PBMC from an ARQ/VRQ sheep (4545) never exposed to scrapie were used as positive and negative controls, respectively. We have previously demonstrated the presence of prions in the blood of sheep 4124 and 4125 by bioassay [8, 9]. Frozen PBMC pellets were thawed on ice and resuspended at 5 × 10^5^ cells/μL in conversion buffer [PBS supplemented with 150 mM NaCl, 4 mM EDTA, 1 % Triton X-100, and complete protease inhibitor (Roche, Nutley, NJ)]. PMCA reactions were seeded by adding PBMC (~1 × 10^7^ cells in 20 μL) to 0.2 mL thin-walled reaction tubes containing 80 μL 10 % normal brain homogenate (mNBH) prepared from scrapie-free Tg338 mice. All samples were assayed in duplicate. Reaction tubes were placed in a microplate horn sonicator (Misonix S-4000; Qsonica) containing liquid coolant diluted in ultra-pure water (Koolance; 30 % v/v) maintained at 37 °C by a recirculating chiller (Solid State Cooling Systems) with cycles of sonication (ultrasonic amplitude of 70 yielding 180–190 W for 30 s) and incubation (29 min 30 s). At the end of 48 cycles, defined as one round, samples were diluted 1:3 into fresh mNBH before starting the next round of PMCA; remaining product was stored at −80 °C. Up to six rounds of PMCA were performed for each sample. sPMCA end-point was detection of the proteinase K resistant misfolded prion protein core (PrP^res^) by western blotting.

sPMCA products generated from PBMC of groups 2 and 3 along with the PBMC isolated from known scrapie-infected VRQ/VRQ sheep (4124 and 4125) [8, 9] and scrapie-uninfected ARQ/VRQ sheep were analyzed by western blot as previously described [13, 14]. Briefly, 10 μl of sPMCA products were diluted 1:2 with 2X lysis buffer (20 mM Tris–HCl (pH 7.5), 1 % NP-40, 1 % sodium deoxycholate) prior to incubation with proteinase K (200 μg/mL final concentration) at 37 °C for 90 min. After addition of NuPAGE LDS sample buffer and NuPAGE sample reducing reagent (Invitrogen, Carlsbad, CA) and boiling for 10 min samples (equivalent to 5.5 μl sPMCA product) were loaded onto a 12 % Nu-PAGE Bis-Tris gel (Invitrogen, Carlsbad, CA). After electrophoresis, proteins were transferred onto PVDF membranes, blocked with commercial casein blocker (Pierce, Rockford, IL) containing 0.05 % Tween 20. Immunodetection of PrP^res^ was accomplished using prion mAb P4 (0.1 μg/ml; R-Biopharm AG, Darmstadt, Germany) and a horseradish peroxidase conjugated secondary Ab (0.05 μg/ml; SouthernBiotech, Birmingham, AL). Peroxidase activity was detected following incubation with enhanced chemiluminescence substrate (Amersham ECL^TM^, GE healthcare, Piscataway, NJ) and exposure to radiographic films (KodakBioMax Chemiluminescence Films).

### Availability of data and materials

The datasets supporting the conclusions of this article are included within the article.
